# Rheological Properties and Inkjet Printability of a Green Silver-Based Conductive Ink for Wearable Flexible Textile Antennas

**DOI:** 10.3390/s24092938

**Published:** 2024-05-05

**Authors:** Abdelkrim Boumegnane, Said Douhi, Assia Batine, Thibault Dormois, Cédric Cochrane, Ayoub Nadi, Omar Cherkaoui, Mohamed Tahiri

**Affiliations:** 1 Organic Synthesis and Extraction Laboratory (OSEV), Ain Chock’s Faculty of Sciences, Hassan II University, Casablanca B.P 5366, Morocco; assiabatine2016@gmail.com (A.B.); mohtahiri@yahoo.fr (M.T.); 2 Textile Materials Research Laboratory (REMTEX), Higher School of Textile and Clothing Industries (ESITH), Casablanca 20230, Morocco; said.douhi.15@gmail.com (S.D.); nadi@esith.ac.ma (A.N.); cherkaoui@esith.ac.ma (O.C.); 3 Laboratory of Physics of Condensed Matter (LPMC), Faculty of Sciences Ben M’Sik, Hassan II University, Casablanca 2000, Morocco; 4 École Nationale Supérieure des Arts et Industries Textiles—ENSAIT, ULR 2461—GEMTEX—Génie et Matériaux Textiles, University of Lille, F-59000 Lille, France; thibault.dormois@ensait.fr

**Keywords:** silver-based ink, inkjet printing, rheological properties, sodium alginate, flexible antennas

## Abstract

The development of e-textiles necessitates the creation of highly conductive inks that are compatible with precise inkjet printing, which remains a key challenge. This work presents an innovative, syringe-based method to optimize a novel bio-sourced silver ink for inkjet printing on textiles. We investigate the relationships between inks’ composition, rheological properties, and printing behavior, ultimately assessing the electrical performance of the fabricated circuits. Using Na–alginate and polyethylene glycol (PEG) as the suspension matrix, we demonstrate their viscosity depends on the component ratios. Rheological control of the silver nanoparticle-laden ink has become paramount for uniform printing on textiles. A specific formulation (3 wt.% AgNPs, 20 wt.% Na–alginate, 40 wt.% PEG, and 40 wt.% solvent) exhibits the optimal rheology, enabling the printing of 0.1 mm thick conductive lines with a low resistivity (8 × 10^−3^ Ω/cm). Our findings pave the way for designing eco-friendly ink formulations that are suitable for inkjet printing flexible antennas and other electronic circuits onto textiles, opening up exciting possibilities for the next generation of E-textiles.

## 1. Introduction

The field of electronic textiles (e-textiles) has experienced remarkable growth in recent years, largely driven by advancements in printed electronics [1]. These advancements have revolutionized the production of flexible electronic circuits and devices, opening up exciting possibilities in sectors such as sportswear [2,3], personal protective equipment (PPE) [4,5], transportation [6], and healthcare [7,8]. This convergence of technology and textiles has given rise to the concept of “intelligent” or “smart” textiles, as efforts are made to seamlessly integrate smart electronic systems into fabrics. However, one of the key challenges in fully realizing the potential of e-textiles lies in creating a reliable textile substrate that can effectively provide electronic functionality while maintaining crucial properties such as flexibility, durability, comfort, and washability [9].

The flexibility of textile substrates plays a crucial role in the integration of wearable electronics and holds significant promise for their application in sensors [10], energy devices [11], and transistors [12], aligning with recent technological advancements. Researchers have been pursuing various design strategies for the integration of electronic components based on their respective disciplines, including printing technologies. Various printing technologies, including flexible lithography [13], flexography [14], screen printing [15], and inkjet printing [16,17], have been employed to manufacture e-textiles. Among these techniques, inkjet printing offers several advantages, including the ability to deposit precise amounts of materials at specific locations on the fabric, providing design freedom. Moreover, inkjet printing is a cost-effective and environmentally friendly production method that can be applied to different surfaces such as paper, polymers, and textiles [18].

The success of wearable electronic textiles hinges on the development of high-performance functional inks [19]. Different types of conductive materials, including metallic nanoparticles [20], carbon nanotubes [21], graphene [22], and conductive polymers [23], have been used as conductive fillers in printed flexible electronics. Metallic fillers, particularly silver nanoparticles (AgNPs), have gained significant attention due to their high conductivity, antioxidant properties, and stability at room temperature. As a result, AgNP-based inks are highly desirable for wearable communication devices, given the ease of incorporating silver into inks [24].

Among the most widely popular portable communication devices, antennas play a crucial role in the design of body-centric wireless systems [25,26]. These systems face the challenge of ensuring optimal power transfer between nodes positioned on various parts of the body, as well as enabling communication with external devices. Recent technological advancements in the field of portable communication systems underscore the importance of developing antennas specifically designed for portability, with mechanical flexibility and efficient radiation as key developmental criteria [27,28]. As a result, the use of silver-based conductive inks in conjunction with inkjet printing technology has set high standards for availability and reliability [29,30]. These advances are at the cutting edge of body-centered communication and meet the growing needs of this field. However, despite the advantages of using conductive silver ink for the development of flexible printed antennas, their widespread use is hampered by challenges such as the complexity of ink preparation processes, the need to balance ink stability and solid content concentration, and achieving an appropriate surface resistance to guarantee antenna efficiency [31].

Achieving high surface electrical conductivity, a requirement for most electronic textiles necessitates a high solid content in the ink. Unfortunately, a high silver content can lead to ink formulation destabilization and clogging of inkjet printer nozzles, posing a significant challenge. To address this, stabilizing polymers are recommended to improve the stability of high-solid-content conductive inks [32,33]. Recent research by Hassan Shahariar et al. demonstrated the development of conductive silver inks for printing conductive traces on polyester fabric, with a silver content ranging from 10 to 30% [34]. However, the high silver content in the inkjet printing formulations led to ink destabilization. In another study conducted by Iara J. et al., a novel formulation of conductive ink was developed, which contained an approximate silver content of 8% [35]. The ink was printed using inkjet technology on PET fabrics and included ethylene glycol as a solvent to prevent nozzle clogging and increase the viscosity of the ink, as well as ethanolamine as a surfactant and emulsifier for ink durability and stability, and a dispersant containing polyesters to enhance ink stability.

Therefore, in light of the specifications of inkjet technology, it becomes imperative to advance the development of conductive silver ink that possesses several desirable characteristics: low silver content, exceptional stability, high conductivity, and reliable performance. Generally, novel formulations of conductive inks typically consist of essential components such as a dispersing polymer, a carrier solvent, additives like surfactants, and a conductive filler [36,37]. Polymers are commonly incorporated into silver inks as stabilizers to prevent the agglomeration of silver nanoparticles (AgNPs). The amount of polymers present in the ink allows for differentiation between liquid ink used specifically for inkjet printing and paste ink utilized for alternative techniques, particularly screen-printing [38]. Consequently, the choice of polymer matrices for silver inks is influenced by the selected printing technique. The ability to perform printing effectively is a crucial factor in determining the appropriate polymer for silver-based conductive inks. When employing low molecular weight polymers, it is typically necessary to include a significant fraction of polymer to confer favorable rheological properties to the ink for easy printing. However, this approach is not recommended from a printing standpoint, as it consistently increases the drying and evaporation time after printing. On the other hand, high-molecular-weight polymers can significantly impede printing quality by introducing undesirable defects during the drying step in inkjet printing. Various types of polymers have been utilized as polymeric matrices in the development of conductive inks for inkjet printing, including both ionic and non-ionic polymers. Among these, polyvinylpyrrolidone (PVP) and Polyethylene glycol (PEG) have been frequently mentioned in the literature due to their dual functionality as dispersants and coating agents [31,37]. Recently, scientists have been actively exploring the synthesis of new polymers derived from sustainable and renewable resources to formulate high-value inks.

Polysaccharide-based polymers have emerged as promising materials for the development of conductive inks due to their abundant availability and environmental sustainability. Additionally, the combination of polysaccharides with PEG has shown potential in creating new polymeric matrices with improved dispersion properties [39]. Conductive ink, being a colloidal solution, exhibits complex rheological behaviors under applied stress or deformation, including shear-thinning effects, yield stress, and thixotropic behavior [40]. Previous studies have demonstrated the dependence of the viscosity of conductive inks on factors such as particle size, particle fraction, and temperature. However, there is limited understanding in the recent literature regarding how the composition of the polymeric matrix influences the rheological properties of inks, especially for inkjet-printed conductive circuits on textile substrates. Therefore, it is essential to conduct research on the rheological behavior of conductive inks under different shear conditions to determine their suitability for the inkjet printing process [41]. These investigations will provide valuable insights and enable the formulation optimization of inks for achieving successful inkjet printing outcomes.

In this study, our main objective was to investigate the rheological properties of silver conductive ink for inkjet technology by using a PEG-doped Na–alginate suspension matrix. Various ink formulations were developed with a silver nanoparticles (AgNPs) content ranging from 0.5% to 3 %wt, employing different Na–alginate/PEG ratios. The inks’ thixotropic and viscosity characteristics were evaluated using a shear-induced rheological model that simulates the printing process. Furthermore, the impact of the ink composition on these properties was investigated to optimize the ink formulation for inkjet printing. Through careful optimization of the ink formulation, an ink highly suitable for inkjet printing applications was successfully developed. Expanding on this achievement, a deeper understanding of the impact of the polymer matrix on the conductive properties of the printed patterns was pursued. Our findings contribute to the advancement of conductive ink technology by highlighting the significance of rheological properties and ink composition in achieving high-quality printed patterns. This knowledge can guide future research and development endeavors aimed at creating improved conductive inks customized for inkjet technology, facilitating their use in various applications such as flexible electronics, printed circuits, and wearable devices.

## 2. Experimental Section 

### 2.1. Material

Polyester fabric (PET, 89.2 g/cm^2^) specifically designed for printing was purchased from ITEX. Sodium alginate (Na–alginate) with high viscosity was purchased from CARLO ERBA, while polyethylene glycol (PEG) with a molecular weight of 300,000 g/mol was supplied from SIGMA ALDRICH. Silver nitrate (AgNO_3_) was provided by VWR chemicals, and sodium carbonate (Na_2_CO_3_) was purchased from FLUKA CHEMICA. Potato starch was acquired from SOLVACHIM. Additional chemical agents, including sodium hydroxide (NaOH), ethanol (CH_3_-CH_2_-OH), and cetyltrimethylammonium (CTAB), were procured from SIGMA ALDRICH. All the chemicals used in this study were of analytical grade. Throughout the experimental work, no additional purification was performed on the mentioned reagents, and deionized water was used for solubilizing the chemicals.

### 2.2. Formulation of Silver-Based Ink

Formulations of conductive inks usually consist of several key components: a dispersing polymer, a vehicle solvent, surfactants as additives, and conductive fillers. These ingredients have a crucial role in enhancing the ink’s properties for printing conductive patterns on various substrates, particularly textiles. The mixture of polymer, solvent, and additives improves the wettability and dispersion stability of conductive particles, allowing the ink to effectively form a film during the printing process. Moreover, careful formulation of these components is vital in adjusting the ink’s rheological properties to ensure optimal performance during the printing process on textile substrates.

To prepare the conductive ink, silver nanoparticles (AgNPs) were synthesized using a simple co-reduction process, following the procedure described in our previous work [42]. Various amounts of AgNP powder ranging from 0.5 %wt to 3 %wt were added to the pre-prepared suspension matrices. The matrices were prepared by combining the Na–alginate solution (3 g/L) with varying proportions of PEG while maintaining a fixed solvent ratio of 3:1 (ethanol: DI water), as illustrated in Table 1.

### 2.3. Rheological Properties of Conductive Inks

Formulated conductive inks underwent rheological characterization using a plate–plate rheometer. To ensure precision, all measurements were repeated three times at room temperature. The shear viscosity of the conductive ink is of vital importance in the inkjet printing process. Therefore, a steady-state rheological test was conducted across a shear rate range from 0 to 300 s^−1^ [41]. Additionally, to understand the ink’s structural changes during printing, its oscillatory rheological properties were examined. This involves subjecting the ink samples to sinusoidal deformation (*σ*), as demonstrated by the following equations:

(1) $$\sigma =\sigma_{o}\;\sin(\omega t)$$

(2) $$\gamma =\gamma_{o}\sin\;(\omega t+\delta )$$

(3) ω=2πf

where *σ_o_* is the stress amplitude, *ω* is the angular velocity, *γ_o_* is the maximum deformation, *δ* is the phase shift, *f* is the frequency, and *t* is the time. In this context, the ratio of the applied shear stress to the deformation is defined as the complex modulus (G*, Equation (4))

(4) $$G^{*}=\frac{\sigma }{\gamma }$$

The complex shear modulus allows for an evaluation of the ability of the conductive ink to withstand deformation during the inkjet printing process. Furthermore, it is possible to express the complex shear modulus in terms of the elastic and viscosity modulus. The modulus of elasticity or storage (*G*′, Equation (5)) is related to the ability of the conductive ink to store energy, and it is the most important parameter used to determine the viscoelastic region. The modulus of viscosity or loss (*G*″, Equation (5)) indicates the fluidity of the conductive ink.

(5) $$G^{*}=G^{\prime }+iG\prime \prime$$

The ratio between *G*′ and *G*″ is referred to as tan *δ* (Equation (6)), which quantifies the balance between energy loss and storage. The tan *δ* factor can represent the damping property of the material and provide insight into the strength of interaction within the internal structure of the conductive ink.

(6) $$\tan\delta =\frac{G\prime \prime }{G^{\prime }}$$

### 2.4. Computational Methodology

The interactions within suspension matrices were investigated through computational chemistry calculations. Calculations were performed using the DFT at B3LYP method with Becke’s three-parameter hybrid exchange function (B3) combined with the Lee–Yang–Parr correlation functional (LYP) [43]. This method was selected for its ability to accurately predict the molecular geometry and vibration modes of medium-sized molecules, even with simple 6–31G (d,p) basis sets, known for their efficiency and time-saving properties. Calculations were carried out using Gaussian 09W 9.5 Revision D.01 software [44]. Interactions were assessed using the Molecular Electrostatic Potential (MEP), which proved to be an effective tool in describing non-covalent interactions, particularly hydrogen bonding [45].

Based on the frontier orbital approach, we examined two crucial quantum parameters of electronic reactivity: *E_HOMO_*, the energy of the highest occupied molecular orbital, and *E_LUMO_*, the energy of the lowest unoccupied molecular orbital, as well as the energy gap (Equation (7))

(7) $$∆E=E_{LUMO}-E_{HOMO}$$

### 2.5. Construction of Conductive Pattern on the PET Fabric

Conductive ink is loaded into a syringe for precise application onto the PU-coated PET surface, as described in our previous work [42]. The syringe’s needle tip is designed to simulate a printer nozzle. This manual printing approach benefits from the ink’s-controlled viscosity, ensuring accurate deposition without absorption. The ink maintains its shape during application, resulting in well-defined patterns. After printing, the ink undergoes a drying process, adhering securely to the PU-coated PET substrate. Upon complete drying, it forms a cohesive layer, ensuring effective electrical conduction. This syringe-based method provides exceptional precision and direct control, facilitating the creation of functional patterns. Our subsequent focus was on evaluating the compatibility of printed PU-coated PET for use in portable communication systems. We tried to demonstrate the practicality of using textiles printed with our innovative ink as adaptable, mobile antennas. To validate this concept, we conducted a thorough validation process, primarily relying on simulation techniques. This comprehensive and systematic evaluation has significantly enhanced our understanding of the extensive application possibilities associated with textiles printed using our specially formulated ink.

### 2.6. Characterization

X-ray diffraction (XRD) measurement of silver nanoparticles was performed on an X-ray diffractometer (The AXS D4 X-ray diffractometer, Brucker, Billerica, MA, USA) using monochromatic Cu Kα radiation (λ =1.54 Ǻ), registered on scale 2θ from 10° to 80°. Thermal analysis of the polymer matrices was carried out through thermogravimetric analysis (TGA) using a Cahn VersaTherm thermogravimetric analyzer, Thermo Fisher, Waltham, USA. The analysis spanned from 25 °C to 800 °C, with sample weights ranging from 5 to 10 mg. The heating rate employed was 10 °C/min, and the analysis was performed under a nitrogen atmosphere with a flow rate of 25 mL/min. To determine the glass transition temperature of the polymer matrix, a DSC131 evo thermal analyzer system (Setaram, Caluire, France) was employed. The DSC instrument was calibrated using metallic indium with a purity of 99.9%. All polymer samples were tested in crimped aluminum pans and subjected to a heating rate of 10 °C/min under a nitrogen flow of 25 mL/min. The temperature range for the analysis was from 20 to 150 °C. Rheological properties of the suspension matrices and silver conductive inks were measured using an RHM01-RD HAAKE-type rheometer (Thermo Fisher, Waltham, MA, USA); all measurements were repeated three times at 25 °C. The surface SEM images of the suspension matrix and printed lines were taken using a scanning electron microscopy Quanta 650 Thermo Fisher Thermo Fisher, Waltham, USA, equipped with an EDS detector (EDAX Instruments Apollo SDD). The optical image of the printed pattern was obtained using the DSX1000 digital microscope (Olympus, Toyo, Japan), equipped with high-resolution objectives. Linear electrical resistance was measured using the MTX 3292 digital multimeter (Metrix, France), following safety standard NF EN 61010-1 + NF EN 61010-2-030. The same leads and probes were used during the measurements. For validation purposes, each reading was repeated three times before the measurement was recorded with the standard deviation value.

## 3. Results and Discussion

### 3.1. Characterization of AgNPs and Suspension Matrices

**Figure 1:**
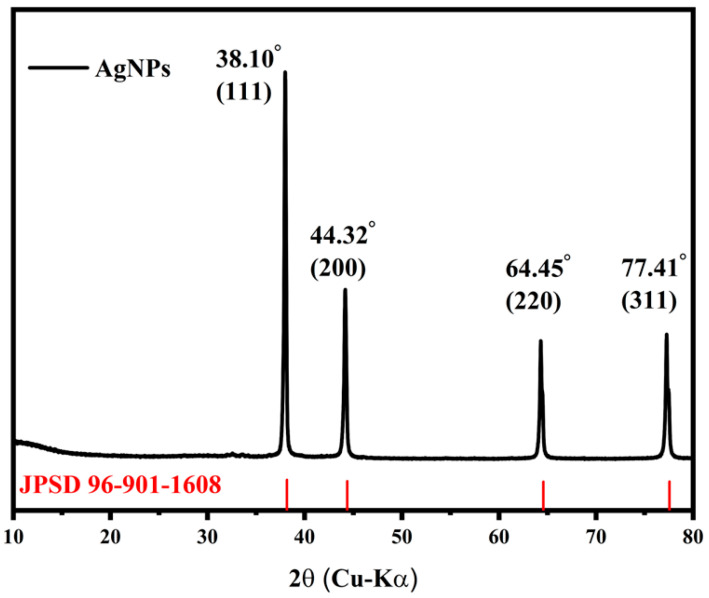
XRD pattern of AgNPs.

The crystal structure analysis of the biosynthesized AgNPs was carried out using the X-ray diffraction (XRD) technique (Figure 1). The resulting XRD diffractogram demonstrates the presence of four distinct peaks located at diffraction angles 2θ of 38.10°, 44.32°, 64.45°, and 77.41°, which can be attributed to the crystal planes (111), (200), (220), and (311), respectively. This correlation is supported by the Crystallography Open Database (COD), entry number JCPDS 96-901-1608.

Determining the particle size is crucial as it is a key parameter in the formulation of conductive inks. Nano-sized particles are widely used in inkjet inks to prevent printhead clogging. XRD analysis can be used to estimate this parameter. To determine the average particle size of AgNPs, the Debye–Scherrer method was used (Equation (8)). Based on the Scherr equation, the average particle size was estimated at 30.85 ± 2.15 nm (Table 2), conforming to the TEM findings [42]. This size meets the requirements of inkjet printing.

(8) $$D=\frac{k\times \lambda }{\beta \;cos\theta }$$

**Figure 2:**
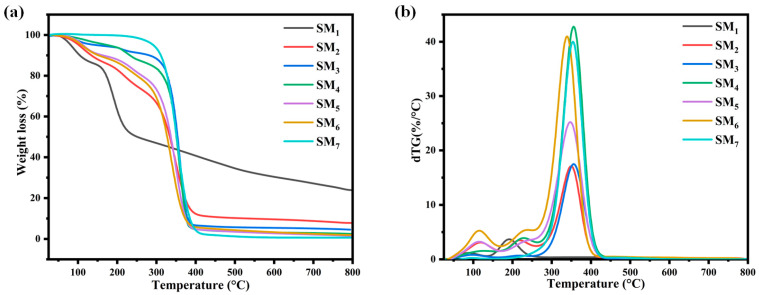
(**a**) TGA and (**b**) DTG curves of suspension matrices.

A thermogravimetric analysis (TGA) was used to evaluate the thermal stability of suspension matrices. The TGA curves of all the samples are presented in Figure 2, while the degradation ranges are summarized in Table 2. The analysis revealed that Na–alginate (SM_1_) undergoes degradation in two stages. The initial stage involves the loss of free water molecules, while the second stage corresponds to decarboxylation, which involves the loss of CO_2_ [46]. As for PEG (SM_7_), the thermogram shows that this later degrades in a single stage within the temperature range of 270–415 °C, with a residue rate of 40%. When Na–alginate is combined with PEG, the thermograms of all of the matrices exhibit three distinct mass loss domains. The first degradation stage corresponds to the loss of water molecules from Na–alginate, with residue percentages ranging from 93 to 97%. The second degradation stage is attributed to the decarboxylation process in the analyzed suspensions, with residue percentages ranging from 80 to 93%. Finally, the third stage represents the degradation of PEG, with residue percentages ranging from 37 to 56%. The degradation stages of the suspension matrices are presented in supplementary data (Table S1). It was observed that an increase in the proportion of PEG in the mixture results in a reduction in the thermal stability of Na–alginate. This can be explained by the formation of intramolecular interactions between PEG and the carboxylate function of Na–alginate, which ultimately affects the overall thermal stability of the mixture, which is similar to what was reported in previous works [47].

**Figure 3:**
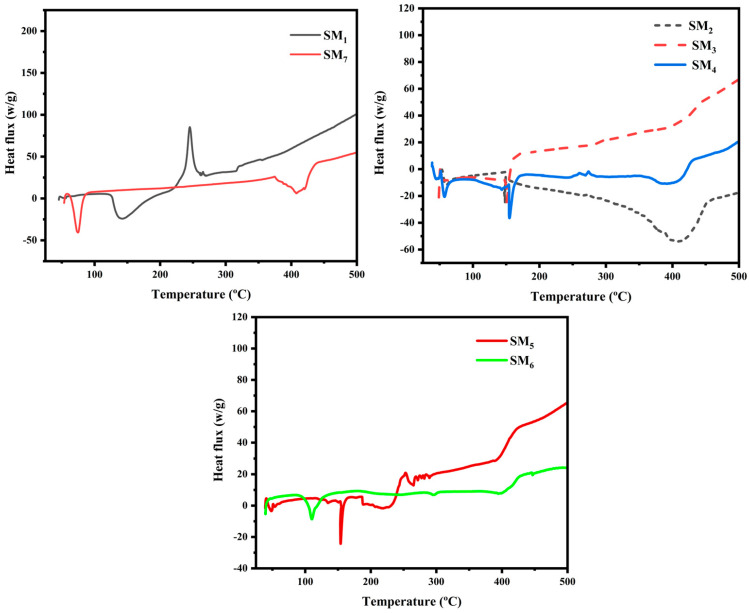
DSC thermograms of suspension matrices.

DSC analysis was used to analyze the inks’ thermal behavior and detect interactions and changes in crystalline structures within suspension matrices. Figure 3 presents the DSC thermograms of the suspension matrices and the obtained results: the Tf (melting temperature) and Td (decomposition temperature) are listed in Table 3.

The SM_1_, composed of pure Na–alginate, exhibits a distinctive thermal profile characterized by two distinguishable peaks. The first endothermic peak is observed at 142 °C, corresponding to the melting temperature and closely resembling the water loss region observed in thermogravimetric analysis (TGA). Na–alginate contains two hydroxyl groups and one carboxylate group that form strong inter- and intramolecular hydrogen bonding, which explains the high value and broad melting peak (Tm).

The second exothermic peak is located at 240 °C, similar to the temperature of the oxidative degradation of polymers [48,49]. As for SM_7_ (pure PEG), its thermogram presents a single endothermic peak at a low temperature of 74 °C, corresponding to the polymer’s melting temperature [50]. Additionally, for all other suspension matrices (SM_2_–SM_6_), two peaks are observed: an endothermic peak similar to the PEG melting temperature, shifting to lower temperature values as the PEG content in the matrices varies, resulting in a reduction in peak intensity. This transition results in a decrease in peak intensity, which can be attributed to the integration of PEG into the Na–alginate system, disrupting the crystalline structure of PEG. Similarly, the second endothermic peak of Na–alginate, resembling the melting temperature, becomes narrower due to interactions between the alcohol functional groups of Na–alginate and the ether groups of PEG. Furthermore, the exothermic peak of Na–alginate is eliminated, allowing us to conclude that the interaction between the carboxyl groups and ether groups of PEG is strong.

### 3.2. Impact of Ink Composition on Rheological Characteristics

**Figure 4:**
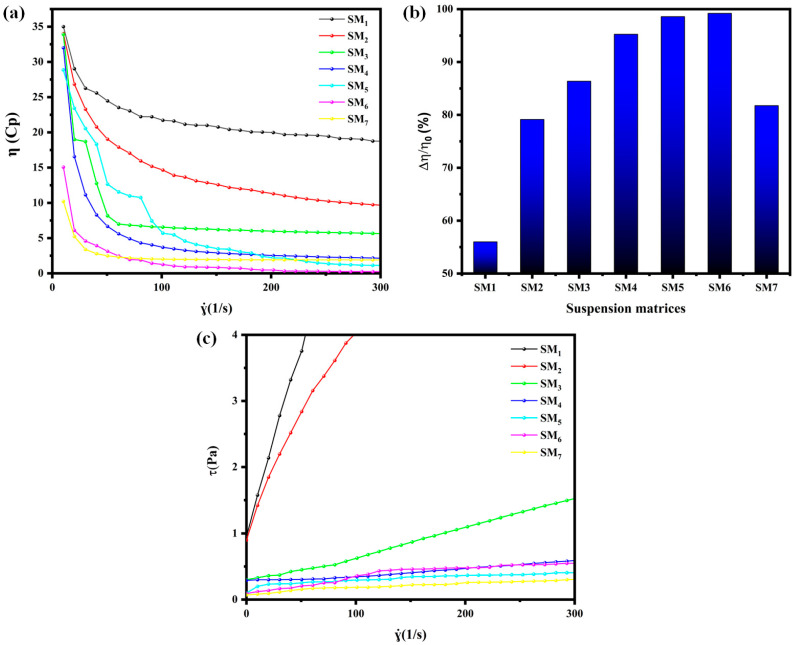
(**a**) Viscosity of suspension matrices at shear rates ranging from zero to 300 s^−1^. (**b**) Loss rate of suspension matrices, and (**c**) shear stress curves of suspension matrices at shear rates ranging from zero to 300 s^−1^.

To investigate the influence of ink formulation on its rheological properties, a study was conducted to analyze the rheological characteristics of the suspension matrices. Subsequently, various amounts of AgNPs as conductive fillers were added to the suspension matrices to produce AgNPs-conductive inks. The viscosity of the seven matrices was analyzed to assess the impact of the Na–alginate/PEG ratio on the matrix flow behavior. Figure 4a shows the viscosity of the suspension matrices at different shear rates. All matrices exhibited thixotropic behavior, demonstrating shear thinning and non-Newtonian flow (rheofluidization) with decreasing viscosity as the shear rate increased.

Increasing the content of PEG had a notable impact on the viscosity of the pure Na–alginate solution, with the most significant decrease being observed in the SM_6_ suspension matrix, where the viscosity reached a minimum of 15 cps. At a zero shear rate, the SM_1_ suspension matrix exhibited a relatively high viscosity of 35 cp. However, as the shear rate was applied, the viscosity of SM_1_ sharply decreased from 0 to 100 s^−1^, followed by a gradual decrease from 100 to 300 s^−1^. This led to a final viscosity of 16.12 cp, resulting in a percentage loss of 54%, as shown in Figure 4b. This change in viscosity behavior can be attributed to the formation of a rigid cross-linked network by the Na–alginate macromolecules through intermolecular and intramolecular hydrogen bonding facilitated by the hydroxyl groups present in the Na–alginate units [51,52]. In contrast, the pure PEG formulation (SM_7_) exhibited an initial viscosity of 10.21 cp, which was 29% lower than that of the pure Na–alginate (SM_1_). Under stress conditions, the viscosity decreased to 1.86 cp, resulting in an 82% loss (Figure 4b). This phenomenon can be ascribed to the flexible molecular structure of PEG, characterized by a lower degree of cross-linking and wider intermolecular spacing.

The incorporation of PEG led to a notable reduction in the viscosity of the suspension matrices. This can be explained by the increased intermolecular distance between the Na–alginate macromolecules caused by the presence of PEG, weakening their interactions. Consequently, the physical entanglement of Na–alginate polymer chains decreased, enhancing their mobility. Matrices SM_2_ to SM_6_ exhibited higher percentage losses compared to formulation SM_1_. For the SM_2_ suspension matrix, its viscosity rapidly decreased up to 150 s^−1^, reaching a final value of 7.31 cP with a percentage loss of 79%. This result can be attributed to the presence of 10% PEG, which improved the mobility of polymer chains. The flow curves of formulations SM_3_ to SM_6_ also showed an immediate decrease between 0 and 100 s^−1^, followed by stabilized viscosity values of 4.78 cp, 1.58 cp, 0.41 cp, and 0.12 cp, respectively.

Without PEG, the dominant molecular interactions within the system were attributed to intramolecular and intermolecular hydrogen bonding in Na–alginate. The dense molecular spacing and strong intermolecular forces impeded the mobility of Na–alginate molecular chains, consistent with the rheological behavior of SM_1_. However, when an appropriate amount of PEG (above 10%) was introduced, the increased intermolecular spacing of the Na–alginate and reduced strength of the hydrogen bonding interactions resulted in the decreased viscosity of the Na–alginate/PEG network. Consequently, the molecular chains within the Na–alginate exhibited enhanced mobility under shear stress due to the presence of a flexible polymer network [51,53].

Figure 4c illustrates the shear stress observed in the formulated suspension matrices. The Figure indicates that all matrices display the characteristics of a viscous fluid flow. Notably, there is a non-zero shear function *τ* (*γ*) at the origin, signifying that our matrices adhere to the Herschel–Bulkley rheological model. This behavior results from the inclusion of a rigid polymer (Na–alginate) within these matrices. The presence of this specific polymer establishes a stable microstructure when the matrices are at rest, giving them rigid properties. However, when subjected to a low force, these matrices exhibit a fluid behavior, underscoring their inherent fluid nature. The rheological equation of the Herschel–Bulkley model is defined by three constants: *τ*_0_, *k*, and *n* (Equation (9)).

(9) $$\tau =\tau_{0}+{k\dot{\gamma \;}}^{n}$$

(10) $$\log(\tau -\tau_{0})=log(k)+nlog\dot{\left(\gamma \right)}$$

where *τ*_0_ represents the threshold stress (Pa), *k* is the consistency index, and *n* is the flow behavior index. It has been demonstrated that the shear stress of the Na–Alginate solution decreases with the incorporation of PEG. These observations reinforce our previous explanations regarding the impact of PEG on Na–Alginate. The *τ* = *f* ($\dot{\gamma }$) curve in the steady-state regime was extrapolated to low shear rate gradients using the linearization method (Equation (10)) to determine the Herschel–Bulkley rheological parameters for the suspension matrices and the results are listed in Table 4. It was found that as the PEG content increases, the values of *τ*_0_ decrease. Matrices SM_5_ and SM_6_ exhibit the lowest values of threshold stress which are 0.1 Pa and 0.09 Pa, respectively, indicating the best flow properties. The value of *n* < 1, suggests that, even with the introduction of the PEG network, the Na–Alginate/PEG matrices remain in the thinning plastic fluids. On the other hand, the values of *k* indicate that the addition of PEG enhances the flow of the mixture. Considering these results and our previous findings, it can be concluded that the addition of PEG significantly reduced the viscosity of the Na–alginate solution and facilitated the flow of the vehicle. This is a significant observation for the development of conductive ink, as the rheological parameters of the suspension matrix composition play a crucial role in determining its flow quality.

### 3.3. Effect of PEG Content on the Dynamic Viscoelasticity of Suspension Matrices

**Figure 5:**
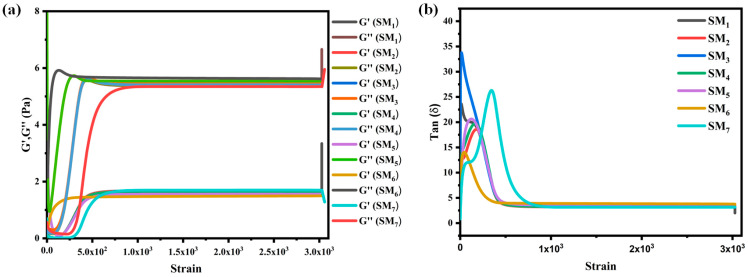
(**a**) Storage and loss modulus and (**b**) tan *δ* factor as a function of deformation for different matrices.

Oscillatory rheological tests were also conducted on the suspension matrices to determine the storage (*G*′) and loss (*G*″) rheological moduli. Figure 5a displays the *G*′ and *G*″ curves of the matrices as a function of deformation. All samples exhibited *G*″ > *G*′, indicating their viscoelastic liquid properties [40].

The oscillatory response revealed two distinct regions: the first region (0–195) displayed low *G*′ and *G*″ values, suggesting the vehicles could withstand mechanical deformation without damaging their macromolecular structures. In contrast, the second region (195–3000) exhibited high *G*′ and *G*″ values (still *G*″ > *G*′), which can be attributed to the transition of formulations into a liquid behavior [54]. According to Figure 5a, it is evident that the *G*′ and *G*″ parameters of the vehicles increased with deformation. This increase was due to there being an insufficient amount of time for the Na–alginate macromolecular chains to disentangle and constrain their orientation along external forces. This observation confirms that the vehicles behave as viscoelastic fluids [55]. Furthermore, the *G*′ values decreased with an increase in the PEG content in the matrices due to the higher proportion of the flexible PEG network, which improved the molecular spacing between Na–alginate macromolecular chains, resulting in a gradual reduction in the degree of interaction. Consequently, it became easier to orient under external forces, leading to irreversible deformation. The viscoelastic behavior of the conductive ink was further analyzed using the tan (*δ*) factor, which characterizes the strength of the interaction within the internal structure of the matrices. Figure 5b illustrates the variation of tan (*δ*) as a function of deformation. All tan (*δ*) values were greater than 1, confirming the viscoelastic liquid characteristics of the vehicles. 

### 3.4. DFT Study of Na–alginate and PEG Interaction 

#### 3.4.1. HOMO-LUMO Analysis

**Figure 6:**
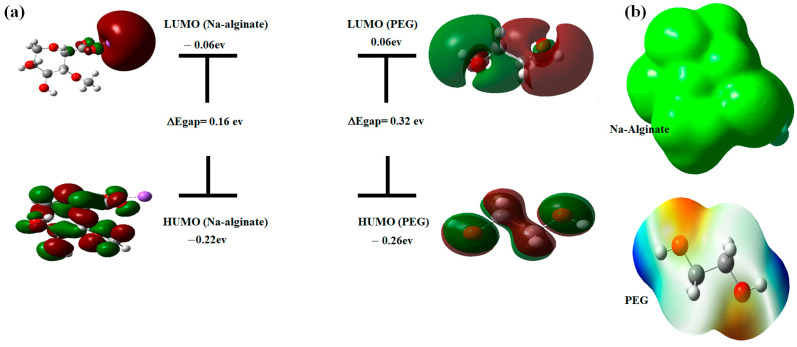
(**a**) ΔEHOMO-LUMO energy gap for Na–alginate and PEG. (**b**) Molecular electrostatic potential (MEP) map.

An analysis of the frontier molecular orbitals was conducted to provide a deeper understanding of the electronic properties within the structures of the Na–alginate and PEG. Isosurfaces representing the highest occupied molecular orbitals (HOMOs) and the lowest unoccupied molecular orbitals (LUMOs) are depicted in Figure 6a. A higher energy difference between HOMO and LUMO orbitals (E_gap_) generally indicates that the molecule tends to be stable and resistant to intramolecular interactions. This suggests that the electrons are strongly localized in specific orbitals, limiting their interactions with other surrounding molecules [56,57]. The energy values of the HOMO (Na–alginate) and LUMO (Na–alginate) orbitals, as well as the HOMO (PEG) and LUMO (PEG) orbitals, are −1.96 eV, −5.20 eV, −0.26 eV, and 0.06 eV, respectively. The energy gap between HOMO and LUMO was determined to be 3.24 eV and 0.32 eV for Na–alginate and PEG, respectively.

Considering the significant energy difference observed for Na–alginate, it is possible to conclude that direct interactions between Na–alginate and PEG chains are less probable. However, it is still conceivable that interactions such as hydrogen bonding to the Na–alginate surface may occur. Furthermore, it is important to note that while the HOMO and LUMO energy gap (E_gap_) values for Na–alginate and PEG give a qualitative insight into the potential interactions between these polymers, they offer only a partial view of the situation.

#### 3.4.2. MEP Mapping 

The molecular electrostatic potential has been proven to be very effective in describing non-covalent interactions, particularly hydrogen bonding. The MEP is widely used as a reactivity map, displaying the most likely region for nucleophilic and electrophilic attacks. On the MEP surface, the color red corresponds to an electron-rich (negative) region, blue indicates an electron-poor (positive) region, and green represents a neutral electrostatic potential. On most MEP surfaces, the negative region is the preferred site for electrophilic attack, while the positive region is favored for nucleophilic attack. Electron concentrations on the MEP surface are indicated by different colors. The electron density values increase in the following order:

Red > Orange > Yellow > Green > Blue

In Na–alginate, the carbon and oxygen atoms are mainly represented by the green color, indicating a null electrostatic potential (Figure 6b). This may suggest that Na–alginate has areas of relatively low electron density. As for PEG, the structure shows both electron-rich zones (represented by colors ranging from yellow to orange and red) and electron-poor zones (represented in blue). The electron-rich zones are located mainly around the electron pair on the oxygen atoms and may indicate a high electron density in these regions. These observations suggest that PEG has regions where electrons are more available for interaction, while Na–alginate appears to have regions with a lower electron density. Combining this information, it is possible to speculate that interactions between Na–alginate and PEG could be influenced by differences in electron distribution. For example, the electron-rich regions of PEG could interact with the less electron-dense regions of Na–alginate, creating electrostatic interactions or hydrogen bonds [56].

### 3.5. Effect of AgNPs on Suspension Matrices Viscosity

**Figure 7:**
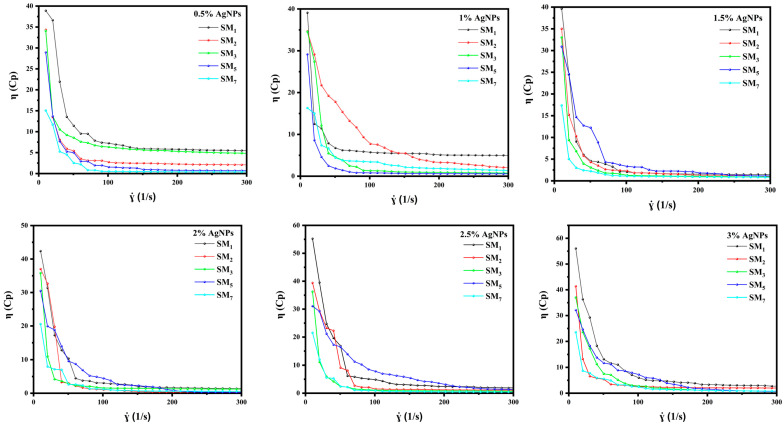
Viscosity of silver conductive inks at shear rates ranging from 0 to 300 s^−1^ with silver filler content ranging from 0.5% to 3%.

Achieving a suspension of AgNPs in a carrier polymer matrix, with suitable rheological properties, is a critical task in the formulation of printable conductive inks. In this section, the rheological behaviors of the conductive inks were examined. Five specific suspension matrices (SM_1_, SM_2_, SM_3_, SM_5,_ and SM_7_) have been carefully selected as carrier polymer matrices. Within these matrices, varying concentrations of AgNPs, ranging from 0.5 %wt to 3 %wt, are systematically dispersed. As can be seen in Figure 7, all five formulations exhibited thixotropic properties, which means they exhibited shear thinning and non-Newtonian characteristics. According to our findings, SM_1_ and SM_2_ exhibited higher viscosities values. However, incorporating AgNPs into these matrices significantly increased the viscosity, which affected their suitability for inkjet printing technology. This increased viscosity can be attributed to several factors:

- Additional Resistance: The presence of solid nanoparticles introduces additional resistance to the movement of the ink formulation, contributing to higher viscosity.
- Flocculation: AgNPs have a high Hamaker constant, representing van der Waals interactions between nanoparticles, which can lead to a phenomenon called flocculation. As the silver content increases, the flocculation effect becomes more noticeable, explaining the increase in viscosity at a zero shear rate.

However, the SM_3_, SM_5_, and SM_7_ formulations exhibited suitable rheological properties for inkjet printing due to the PEG effect which led to a reduction in the rigidity of the Na–alginate polymer matrix. Among the five inks, SM_5_ was found to be the best choice for use as conductive ink in inkjet printing. This formulation provided good dispersion without compromising its viscosity. This makes it suitable for precise printing applications requiring stable conductive inks [58,59].

### 3.6. Effect of AgNPs on the Flow Capacity of Vehicles

**Figure 8:**
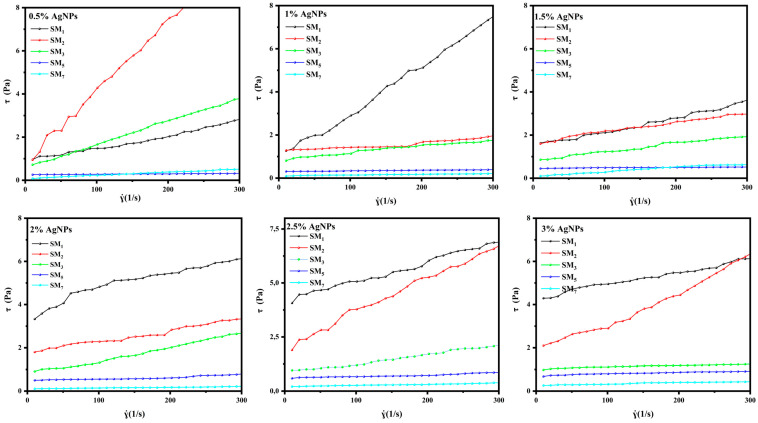
Shear stress curves of silver conductive inks at shear rates ranging from zero to 300 s^−1^ with AgNPs content ranging from 0.5% to 3%.

Figure 8 presents the shear stress as a function of the shear rate for conductive inks. Based on the curves, it is clear that the initial value of shear stress is non-zero, indicating that our inks follow the Herschel–Bulkley rheological model. Additionally, it was observed that the yield strength of the inks increases with an increase in the AgNPs amount. This is because AgNPs create rigid networks as a result of Van der Waals interactions between them and the suspension matrix. Regarding SM_1_, the addition of AgNPs significantly increases its yield strength. This is because the Na–alginate chains, along with the nanoparticles, form a system that is difficult to break down. For the other formulations, lower shear stresses are observed compared to SM_1_. This can be attributed to the presence of PEG, which reduces interactions between Na–alginate chains and AgNPs, helping to reduce the shear stresses.

**Figure 9:**
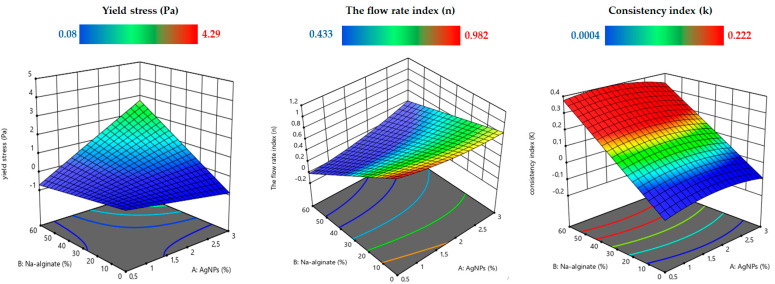
The Herschel–Bulkley rheological parameters (yield stress (*τ*_0_), flow index (*n*), and consistency index (*k*)) for the silver conductive inks.

Figure 9 shows the effect of ink composition on rheological parameters. It was observed that the addition of AgNPs significantly increased the yield stress (*τ*_0_), flow index (*n*), and consistency index (*k*), which was mainly attributed to the inherent viscosity of AgNPs. The detailed experimental findings are accessible in the supplementary data (Table S2). Notably, all flow index values consistently registered below unity (*n* < 1) in the experimental results, conclusively establishing that our conductive inks maintain a thin, plastic, fluid nature. Moreover, the evaluation of the consistency index further underscores the favorable flow behavior of the conductive inks. Notably, the SM_5_ formulation exhibits superior flow behavior, as evidenced by its considerably lower “*k*” values. This performance advantage suggests that SM_5_ holds promising potential for inkjet printing applications requiring excellent flow properties [60].

### 3.7. Electrical Properties of Conductive Ink and Printed Conductive Lines

**Figure 10:**
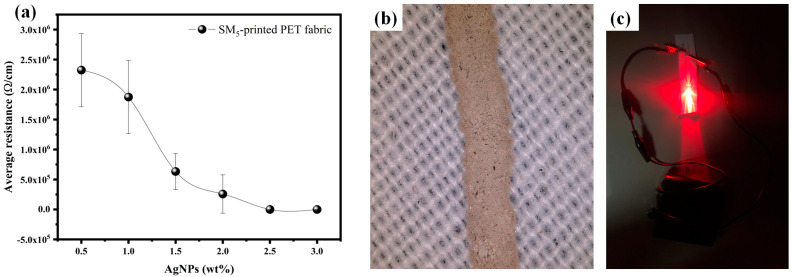
(**a**) Variation in resistance as a function of AgNPs wt% for the SM_5_ formulation, (**b**) optical image of the printed sample, and (**c**) LED test using the printed sample.

It is well known that viscosity and rheological appearance are crucial properties for classifying inks into two categories: low-viscosity inks and high-viscosity ones. Low-viscosity inks are generally used for inkjet printing, with a viscosity ranging between 13 and 30 mPa.s [61,62]. From the rheological results, it is clear that despite the addition of 3 wt% AgNPs to SM_5_, its viscosity value remains within the recommended range for inkjet ink, making it the most suitable ink for inkjet printing. Based on this finding, we delved into the electrical characteristics of the printed patterns made using this chosen formulation with varying amounts of AgNPs. The printing was carried out using a syringe deposition system. This technique allows rapid prototyping by creating electronic patterns directly on textiles. The accuracy of the printed line’s thickness was maintained at 0.1 mm, measured using a highly reliable micrometric screw gauge. After the printing process, we conducted precise electrical assessments. Through a systematic measurement of resistance relative to AgNP concentration, a discernible relationship between the printed pattern’s resistance and the AgNPs weight percentage was established, as presented in Figure 10a. Fabrics printed with 0.5 wt% AgNPs exhibited non-conductivity, as the insufficient AgNP content prevented the formation of an effective conductive path. However, an increase in AgNP content from 0.5 wt% to 3 wt% induced a substantial decrease in resistance, from 2.325 MΩ/cm to 0.008 Ω/cm. This decreasing effect is mainly due to the increase in AgNP content, which enhances the formation of an efficacious conductive pathway. The lowest value is sufficient to switch on an LED connected to the printed PU-coated PET.

Meanwhile, a systematic characterization of the conductive ink’s morphology was carried out to establish the relationship between its formulation and its conductive properties. Scanning Electron Microscope (SEM) images of the SM_1_ and SM_5_ are presented in Figure 10a,b. For the Na–alginate-based matrix (MS_1_), it is clear from the SEM image (Figure 10a) that it has a compact, dense structure with low porosity. These properties lead to it having a limited molecular mobility, justifying its high viscosity value and confirming our findings from the rheological study.

For SM_5_ formed from Na–alginate doped with PEG, it is evident from the SEM image (Figure 10b) that the morphology reveals disorganization due to the incorporation of PEG into the Na–alginate matrix, leading to larger pores [63]. This observation allowed us to justify the significant difference in viscosity between SM_1_ and SM_5_, consistent with the results of the rheological measurement. The increase in pore size results in a reduction in viscosity, which improves molecular mobility within the SM_5_ matrix and potentially facilitates ink flow. This reduction in viscosity makes it highly compatible with inkjet printing, especially when optimized rheological parameters are taken into account. As a result, the SM_5_ formulation has better flow characteristics through the syringe needle, resembling a printhead.

**Figure 11:**
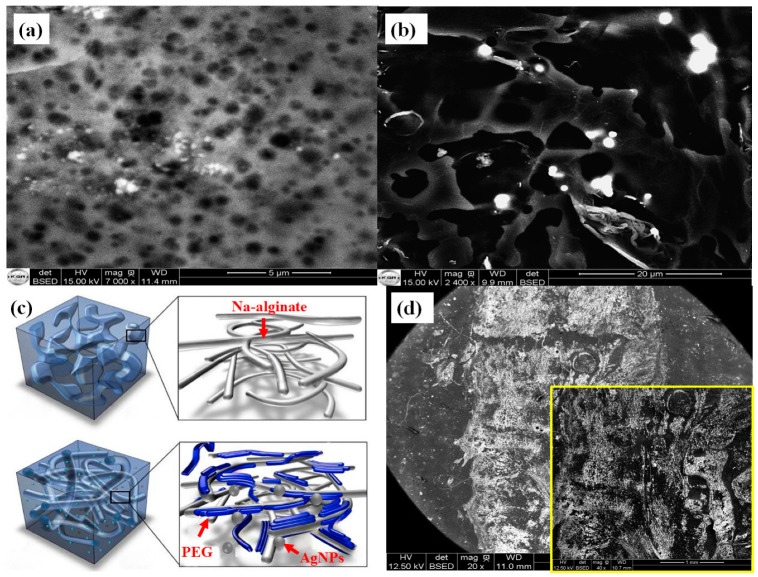
(**a**,**b**) SEM images comparing SM_1_ with SM_5_ based silver ink. (**c**) Illustration demonstrating PEG incorporation into the Na-alginate network during AgNPs ink preparation, and (**d**) SEM image of PU-coated PET printed with SM_5_ conductive ink (1 mm magnification in the yellow box).

In addition to its effect on reducing viscosity, we can observe that the pores within the SM_5_ matrix provide specific sites for the suspension of AgNPs, as illustrated in Figure 11b,c. Due to their small size, the AgNPs disperse evenly within these gaps, providing a homogeneous distribution throughout the matrix. This uniform dispersion of AgNPs increases the available pathways for electrical conduction within the matrix, leveraging the inherent conductive properties of AgNPs. Consequently, the overall electrical resistance of the conductive ink decreases, even when using relatively low silver concentrations (3 %wt). Figure 11d shows the SEM image of the conductive line printed with the SM_5_ formulation containing 3% AgNP. It can be seen that the printed conductive track has sharp ends with well-defined edges and fewer holes along the conductive path, which significantly improves the conductive properties of the printed ink. In summary, the appropriate rheological properties of the SM_5_ matrix enable the production of a high-quality conductive line, making the material exceptionally suitable for a variety of applications requiring efficient electrical conductivity [64].

### 3.8. Potential Application of Silver-Based Ink in Wearable Textile Antennas

To investigate the suitability of SM_5_ printed PET fabric as an antenna for wearable electronics devices, an extensive computational analysis was conducted. The aim was to predict the behavior of this antenna and identify any deviation when it was placed on the body. Ansys 2020 R1 - High-Frequency Structure Simulator (HFSS) software, based on the finite element (FEM) method, was used to optimize the dimensional parameters and analyze the antenna’s electromagnetic properties. The PET material employed in the study possessed specific characteristics that included a relative permittivity (*ε_r_*) of 1.56, a loss tangent (tan *δ*) of 0.027, and a substrate height of 1.5 mm [65]. This section focuses on designing a passive and flexible rectangular microstrip antenna, utilizing PET fabric printed with silver-based conductive ink with 3 %wt of AgNPs (SM_5_), characterized by a resistance of 0.008 Ω/cm and a conductivity of 1.25 × 10^6^ S/m.

The operational characteristics of the antenna were subsequently assessed through simulations. The proposed antenna design takes a multidisciplinary approach, combining electromagnetic engineering with materials science. The antenna’s plate dimensions were determined using Equations (11) and (12) from the transmission line model during the microstrip antenna design process [66,67].

(11) $$W_{p}=\frac{c}{2f_{0}}\sqrt{\frac{2}{\varepsilon_{r}+1}}\;$$

(12) $$L_{P}=\frac{c}{2f_{0}\sqrt{\varepsilon_{eff}}}-2\Delta L$$

where *W_p_* and *L_p_* represent the patch’s width and length, respectively, and *ε_eff_* and *ε_r_* are the effective and relative permittivity of a substrate. The parameters *c*, *f*_0_, and *ΔL* represent the speed of light, central frequency, and effective length.

**Figure 12:**
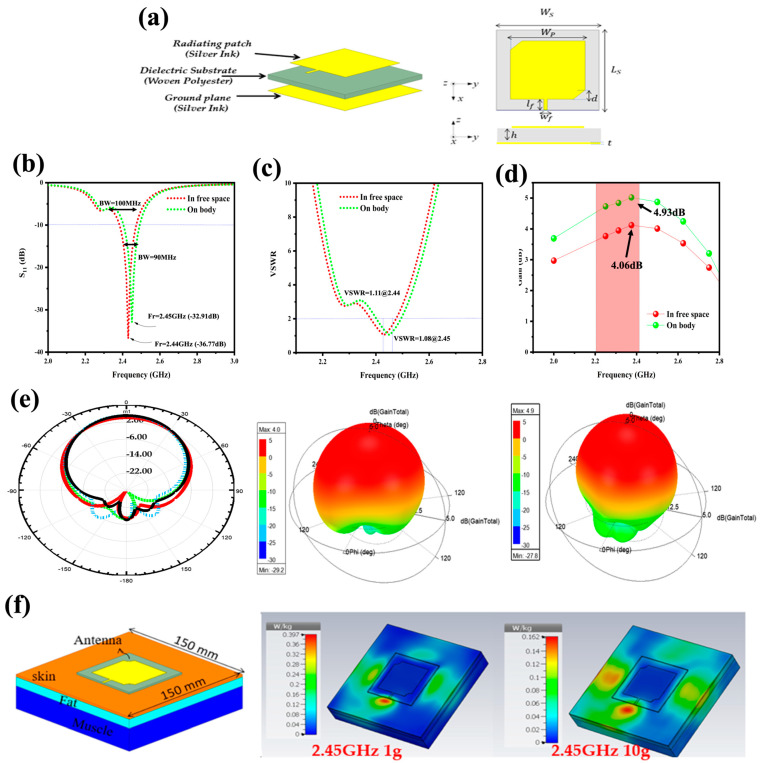
(**a**) Depiction of the configurations of the proposed microstrip flexible antenna in perspective view and top view. Simulated results for both scenarios (Free Space and On-Body): (**b**) reflection coefficient (S11), and (**c**) voltage standing wave ratio (VSWR). (**d**) Maximum gain (the pink bar indicates the highest gain within the antenna’s operating bandwidth). (**e**) Two-dimensional planar gain in free space and when the antenna is located within the human body (Black line: On body (Φ = 0)), and three-dimensional directional gain. (**f**) Visualization of the proposed antenna placement on human tissue in CST studio suite and associated SAR distribution.

The application involves integrating fabric microstrip antennas into clothing. The configuration and simulated dimensions of the fabric antenna are illustrated in Figure 12a. The optimized dimensions of the PET substrate are 70 mm × 70 mm, while the patch length (*l_p_*) and patch width (*W_p_*) of the antenna’s radiating element (silver-based conductive ink (SM_5_)) are 42.50 mm and 43.25 mm, respectively. The width of the feedline (*W_f_*) is 3.85 mm, which allows the microstrip feedline to maintain a characteristic impedance of 50 Ω over the designated frequency range. Additionally, the length of the feedline (*l_f_*) is 7 mm. The proposed antenna utilizes a basic microstrip feedline feeding mechanism instead of a coaxial probe, aiming to minimize protrusions and improve usability.

The simulated reflection loss values (S_11_) for the fabric antenna were examined both in free space and when placed on the body, as shown in Figure 12b. For the antenna operating in free space, resonance occurred at 2.44 GHz with a bandwidth of 100 MHz. However, when the antenna was placed on the body, the resonance shifted slightly to 2.45 GHz, and the bandwidth was reduced to 90 MHz. This slight reduction in bandwidth could be understood from the perspective of the body’s dissipative nature. Furthermore, the reflection losses remained consistently less than 10 dB in both situations. This suggests that the antenna’s ability to reflect energy while transmitting electromagnetic wave signals is significantly reduced.

Another crucial parameter to consider in antenna design is the VSWR (Voltage Standing Wave Ratio), which is a measure used to assess the matching of an antenna or transmission system to a transmission line. It indicates how well the antenna’s impedance matches the characteristic impedance of the transmission line. Figure 12c demonstrates that the antenna exhibits better adaptation performance, with a VSWR lower than two at the operating frequency in both free space and when placed on the human body. Figure 12d illustrates the variation of the antenna’s gain with frequency in the two situations. The antenna operating in free space achieves a maximum gain of 4.06 dB, while the antenna on the body reaches a gain of 4.93 dB. This slight gain increase could also be understood from the perspective of the body’s dissipative nature [68,69].

The radiation pattern serves as a visual representation that depicts how radiated energy is distributed in space. This diagram plays a crucial role in assessing an antenna’s overall performance. Figure 12e showcases the two-dimensional planar gain and three-dimensional directional gain of the antenna, aiming to evaluate its radiation characteristics in both free space and when integrated into the human body. For each frequency, the Figure provides insights into the E-plane (electric field) at an angle ϕ = 90° and the H-plane (magnetic field) at an angle ϕ = 0°. As illustrated, the simulation results for the radiation pattern reveal a prominent main lobe in the desired direction, accompanied by a smaller back lobe in the opposite direction. This observation highlights the antenna’s favorable electromagnetic performance, confirming its suitability for practical applications.

Assessing the Specific Absorption Rate (SAR) is crucial to determine whether the antenna has induced radiation effects on the human body. A diagram depicting the SAR test model is shown in Figure 12f. In this representation, the simulated human model consists of three layers: skin, fat, and muscle. The respective thicknesses of these tissue layers are 2 mm, 8 mm, and 20 mm, resulting in a total human model thickness of 30 mm. To streamline the computation time and enhance the visualization clarity, the dimensions of the human phantom were simplified to 150 mm × 150 mm. The calculation employs an input power of 0.5 W. The electrical properties at 2.45 GHz and the thickness of the human tissue are listed in Table 5. 

The SAR value must remain below 1.6 W/kg per gram of tissue as per the US standard and below 2 W/kg per 10 g of tissue as per the EU standard [4]. We conducted simulations to determine the SAR value at a frequency of 2.45 GHz. Furthermore, Figure 12f illustrates the three-dimensional SAR distribution for the proposed antenna design. The maximum SAR values according to the 1 g and 10 g standards are 0.397 W/kg and 0.162 W/kg, respectively, both falling within FCC’s permissible limits [71,72].

Based on these findings, the designed antenna exhibits an efficient performance at 2.45 GHz, a frequency range widely employed for wireless communication technologies such as WLAN and Bluetooth [73,74]. These results affirm the viability of employing flexible silver-based antennas as a seamlessly integrated, transparent communication interface within textiles. This advancement holds the promise of replacing current rigid and constraining approaches, known for their limitations and adverse environmental effects. It marks a notable stride towards a future guided by ecological awareness and a commitment to sustainability.

## 4. Conclusions

In summary, conductive inks based on silver nanoparticles at a concentration of 3% wt., combined with a variable mixture of Na–alginate and PEG, have been developed. The focus was on understanding the effects of the suspension matrix composition on the rheological behavior of the ink, which then influences its printing onto coated PET fabric. The results of flow and deformation tests demonstrated the importance of PEG quantity in controlling the suspension flow. In addition, the quality of the surface and the sharpness of the lines printed on the fabric are closely linked to the rheological properties of the ink.

The intermediate formulation, called SM_5_, containing 20% Na–alginate and 40% PEG by weight, showed the best flow properties and optimum print quality, as confirmed by Scanning Electron Microscope analysis. The favorable rheological properties of this formulation in the range recommended for the inkjet printing process explain these results. Thanks to this formulation, the electrical resistivity of the resulting e-textile reached only 8 × 10^−3^ Ω/cm, despite a thickness of just 0.1 mm. In addition, conductive textiles printed with this ink demonstrated strong potential as antennas, as predicted by simulations. The promising results obtained with the SM_5_ formulation indicate that conductive inks developed with precise control over their composition are well suited to e-textile applications, particularly as flexible antennas (2.45 GHz). This innovation offers a practical alternative for fully integrated communication in textiles, capable of substituting current methods that are rigid, restrictive, and harmful to the environment.

## Figures and Tables

**Table 1:** Composition of suspension matrices for AgNPs conductive ink.

	SM_1_	SM_2_	SM_3_	SM_4_	SM_5_	SM_6_	SM_7_
Na–Alginate (%)	60	50	40	30	20	10	0
PEG (%)	0	10	20	30	40	50	60
EtOH: DI (%)	40						

**Table 2:** AgNPs particle size measurement using the Debye–Scherrer equation.

2θ	hkl	β	D (nm)	D Average (nm)
38.10	111	0.251	34.94	30.85 ± 2.15
44.32	200	0.288	31.06	
64.45	220	0.325	30.09	
77.41	311	0.388	2731	

**Table 3:** T_f_ and T_d_ values of the suspension matrices.

Suspension Matrix	T_f_ of PEG (°C)	T_f_ of Na–Alginate (°C)	T_d_ of Na–Alginate (°C)
SM_1_	--	142	240
SM_2_	61	148	--
SM_3_	55	151	--
SM_4_	59	156	--
SM_5_	48	153	--
SM_6_	49	120	--
SM_7_	74	--	--

**Table 4:** The Herschel–Bulkley rheological parameters for the suspension matrices.

Suspension Matrix	*τ*_0_	*n*	Log *k*	*k*
SM_1_	0.95	0.820 ± 0.003	−0.848 ± 0.009	0.141
SM_2_	0.90	0.743 ± 0.002	−0.995 ± 0.006	0.101
SM_3_	0.30	0.380 ± 0.018	−0.572 ± 0.031	0.267
SM_4_	0.29	0.668 ± 0.114	−2.914 ± 0.187	0.001
SM_5_	0.10	0.615 ± 0.020	−1.925 ± 0.053	0.011
SM_6_	0.09	0.768 ± 0.012	−2.219 ± 0.033	0.006
SM_7_	0.07	0.775 ± 0.010	−2.520 ± 0.028	0.003

**Table 5:** Characteristics of the human body model’s material properties [25,70].

	Skin	Fat	Muscle
Permittivity	37.95	5.27	52.67
Density (Kg/m^3^)	1001	900	1006
Conductivity (S/cm)	1.49	0.11	1.77
Thickness (mm)	2	8	10

## Data Availability

The authors confirm that all data generated or used during the study are available in the article and the Supplementary Materials.

## Supplementary Materials

The following supporting information can be downloaded at: https://www.mdpi.com/article/10.3390/s24092938/s1, Table S1: Degradation of suspension matrices.; Table S2: The Herschel-Bulkley rheological parameters (yield stress (*τ*_0_), flow index (n), and consistency index (k) for the silver conductive inks.
