# Supplementary material for: Rheological Properties and Inkjet Printability of a Green Silver-Based Conductive Ink for Wearable Flexible Textile Antennas
Source: Sensors (Basel). 2024 May 5;24(9):2938. doi: 10.3390/s24092938 (PMC11086166; doi:10.3390/s24092938)
Supplement: Supplementary file 1 [file sensors-24-02938-s001.zip › sensors-2962998-supplementary.pdf]

Rheological Properties and Inkjet Printability of a Green Silver-Based Conductive Ink for Wearable Flexible Textile Antennas

Abdelkrim Boumegnane[1, 2*], Said Douhi[2, 3,] Assia Batine[1, 2], Thibault Dormois[4], Cedric Cochrane[4*], Ayoub Nadi[2], Omar Cherkaoui[2] and Mohamed Tahiri[1]

[1]*Organic Synthesis and Extraction Laboratory (OSEV), Ain Chock's Faculty of Sciences, Hassan II University, Casablanca B.P 5366 20100, Morocco.*

[2] *Textile Materials Research Laboratory (REMTEX), Higher School of Textile and Clothing Industries (ESITH), Casablanca 20230, Morocco*

[3]*Laboratory of Physics of Condensed Matter (LPMC), Faculty of Sciences Ben M'Sik, Hassan II University, Casablanca 2000, Morocco*

[4] *École Nationale Supérieure des Arts et Industries Textiles—ENSAIT, ULR 2461—GEMTEX—Génie et Matériaux Textiles, University of Lille, F-59000 Lille, France*

*Corresponding author: boumegnanechimie.96@gmail.com (A.B.); cedric.cochrane@ensait.fr (C.C.)

Supplementary data

Table S1: Degradation of suspension matrices.

Suspension matrix	Stage I (ºC)	Stage II (ºC)	Stage III (ºC)
SM_1	60-134	144-245	------
SM_2	80-160	181-263	275-399
SM_3	60-155	172-248	270-414
SM_4	82-167	182-265	278-412
SM_5	72-168	180-255	270-402
SM_6	80-163	185-253	255-391
SM_7	------	------	270-415

Table S2: The Herschel-Bulkley rheological parameters (yield stress (τ_o), flow index (n), and consistency index (k) for the silver conductive inks

Silver content (%)	Formulations	Yield stress (τ_o)	Flow index (n)	Consistency index (k)	R^2
0.5	SM_1	0.96	0.551±0.015	0.158	0.94
	SM_2	0.97	0.836±0.009	0.088	0.98
	SM_3	0.72	0.595±0.011	0.145	0.96
	SM_5	0.25	0.895±0.017	0.0004	0.96
	SM_7	0.08	0.936±0.007	0.002	0.99
1	SM_1	1.27	0.776±0.011	0.102	0.97
	SM_2	1.37	0.657±0.050	0.090	0.75
	SM_3	0.81	0.873±0.016	0.007	0.96
	SM_5	0.31	0.958±0.038	0.0007	0.87
	SM_7	0.094	0.644±0.011	0.003	0.97
1.5	SM_1	1.63	0.509±0.015	0.222	0.93
	SM_2	1.60	0.433±0.015	0.206	0.90
	SM_3	0.86	0.454±0.015	0.196	0.92
	SM_5	0.45	0.697±0.032	0.001	0.82
	SM_7	0.102	0.647±0.011	0.012	0.97
2	SM_1	3.33	0.617±0.023	0.123	0.90
	SM_2	1.80	0.478±0.018	0.185	0.90
	SM_3	0.91	0.526±0.015	0.164	0.93
	SM_5	0.50	0.942±0.028	0.001	0.93
	SM_7	0.106	0.982±0.014	0.0004	0.97
2.5	SM_1	4.07	0.664±0.020	0.099	0.92
	SM_2	1.90	0.667±0.014	0.141	0.95
	SM_3	0.96	0.474±0.016	0.176	0.92
	SM_5	0.59	0.768±0.019	0.003	0.94
	SM_7	0.21	0.980±0.010	0.0008	0.96
3	SM_1	4.29	0.628±0.022	0.108	0.90
	SM_2	2,10	0.667±0.014	0.141	0.96
	SM_3	0.98	0.713±0.023	0.006	0.91
	SM_5	0.68	0.582±0.017	0.009	0.92
	SM_7	0.26	0.723±0.020	0.003	0.93
